# Activity of propolis from Mexico on the proliferation and virulence factors of *Candida albicans*

**DOI:** 10.1186/s12866-023-03064-9

**Published:** 2023-11-04

**Authors:** Mario Rodriguez-Canales, Yoli Mariana Medina-Romero, Marco Aurelio Rodriguez-Monroy, Uriel Nava-Solis, Sandra Isabel Bolaños-Cruz, Maria Jimena Mendoza-Romero, Jorge E. Campos, Ana Bertha Hernandez-Hernandez, Yolanda I. Chirino, Tonatiuh Cruz-Sanchez, Carlos Gerardo Garcia-Tovar, Maria Margarita Canales-Martinez

**Affiliations:** 1 Pharmacognosy Laboratory, Biotechnology and Prototypes Unit, Faculty of Higher Studies Iztacala, National Autonomous University of Mexico, Av. de los Barrios No. 1, Los Reyes Iztacala, Tlalnepantla, Edo. de México C.P. 54090 Mexico; 2 Biomedical Research Laboratory in Natural Products, Medicine Career, Faculty of Higher Studies Iztacala, National Autonomous University of Mexico, Avenida de los Barrios Numero 1, Colonia Los Reyes Iztacala, Tlalnepantla, Edo. de Mexico C.P. 54090 Mexico; 3 Molecular Biochemistry Laboratory, Biotechnology and Prototypes Unit, Faculty of Higher Studies Iztacala, National Autonomous University of Mexico, Av. de los Barrios No. 1, Los Reyes Iztacala, Tlalnepantla, Edo. de México C.P. 54090 México; 4 Laboratory 10, Carcinogenesis and Toxicology, Biomedicine Unit, Faculty of Higher Studies Iztacala, National Autonomous University of Mexico, Avenida de los Barrios Numero 1, Colonia Los Reyes Iztacala, Tlalnepantla, Edo. de Mexico C.P. 54090 Mexico; 5 Propolis Analysis Service Laboratory, Faculty of Higher Studies Cuautitlan, National Autonomous University of Mexico, Av. Teoloyucan Km 2.5, San Sebastian Xhala, Cuautitlán Izcalli, Edo. de México C.P. 54714 México; 6 Laboratory of Veterinary Morphology and Cell Biology, Faculty of Higher Studies Cuautitlan, National Autonomous University of Mexico, Av. Teoloyucan Km 2.5, San Sebastian Xhala, Cuautitlán Izcalli, Estado de México CP 54714 México

**Keywords:** Natural antifungal agents, Anti-opportunistic infection, Natural products targets

## Abstract

**Background:**

This research evaluated the anti-*Candida albicans* effect of Mexican propolis from Chihuahua.

Chemical composition of the ethanolic extract of propolis was determined by GC-MS, HPLC-DAD, and HPLC-MS. The presence of anthraquinone, aromatic acid, fatty acids, flavonoids, and carbohydrates was revealed.

**Results:**

The anti-*Candida* activity of propolis was determined. The inhibitions halos were between 10.0 to 11.8 mm; 25% minimum inhibitory concentration (0.5 mg/ml) was fungistatic, and 50% minimum inhibitory concentration (1.0 mg/ml) was fungicidal.

The effect of propolis on the capability of *C. albicans* to change its morphology was evaluated. 25% minimum inhibitory concentration inhibited to 50% of germ tube formation.

Staining with calcofluor-white and propidium iodide was performed, showing that the propolis affected the integrity of the cell membrane.

*INT1* gene expression was evaluated by qRT-PCR. Propolis significantly inhibited the expression of the *INT1* gene encodes an adhesin (Int1p).

Chihuahua propolis extract inhibited the proliferation of *Candida albicans*, the development of the germ tube, and the synthesis of adhesin INT1.

**Conclusions:**

Given the properties demonstrated for Chihuahua propolis, we propose that it is a candidate to be considered as an ideal antifungal agent to help treat this infection since it would not have the toxic effects of conventional antifungals.

## Introduction

*Candida albicans* is a ubiquitous, opportunistic/facultative pathogen that, under normal conditions, is a member of the healthy microbiota of the gastrointestinal tract, reproductive tract, oral cavity and skin of most humans [1]. *C. albicans* is present in the oral cavity of up to 75% of the population [2]. However, mild to heavy immunocompromised hosts can frequently suffer from persistent and strong *C. albicans* infections; thus, the variety of diseases that *C. albicans* can cause ranges from superficial mucosal infections to systemic disorders with a mortality rate of approximately 40% worldwide [3, 4]. Importantly, this highlights that while *Candida* species are human commensal microorganisms, opportunistic fungal infections can occur not only in immunocompromised patients but also in individuals with intact immune systems.

Importantly, *C. albicans* is the most common nosocomial fungal pathogen and one of the most commonly encountered fungi responsible for human disease [3]; with an increase in the number of invasive medical procedures and with a growing population of immunocompromised people, *C. albicans* infections and, to a lesser extent, other *Candida* species infections are becoming increasingly frequent [4].

Not only does the dysfunctional immune system response of the hosts favour the overgrowth of *C. albicans* and the establishment of candidiasis, but *C. albicans* virulence factors themselves are equally determinant of the severity of the infection. The expression of surface molecules, such as the INT1 gene/related adhesin; formation of biofilms; secretion of hydrolytic enzymes; the ability to change its morphology from yeast to hyphal forms; and its metabolic adaptability are some *Candida* virulence factors [4] that allow *C. albicans* to infect diverse host niches [2].

Currently, there are only four classes of antifungals in clinical use: azoles, polyenes, echinocandins and pyrimidine analogues [5, 6]. In this regard, recent studies have been carried out with derivates of azoles or combinations of these [7, 8] to discover and develop novel antifungal agents with efficiency against resistant strains, broad spectrum of action, high potency and low toxicity. Nevertheless, more studies and, importantly, novel alternatives should be addressed to address the strain resistance problem.

The limited set of treatment options is a problem by itself, but it is also combined with the emerging issue of acquired resistance to one or multiple drugs, decreasing the chances of a successful outcome [9]. The emergence of these resistant strains has been increasing in recent years, and furthermore, such resistant phenotypes can develop over the course of an infection and in response to treatment, thus adding another difficulty in candidiasis treatment [10].

In recent decades, natural products and their chemical compounds have been proposed as candidates for the future development of new medicines due to an ample spectrum of therapeutic effects and low toxicity [11]; they can be used as alternative treatments of different diseases due to their wide range of complementary or synergic activities that have similar therapeutic targets and rarely cause adverse effects [12].

Propolis is a resinous product made by bees from different plant exudates or resins and hence a variety of phytocompounds that bees mix with wax and their salivary secretions [13].

Several works have been published in which it has been shown that propolis possesses pharmacological activities such as antioxidant, anti-inflammatory, immunomodulatory, anticancer, antiviral and antifungal activities, among others [13]; these biological properties are directly related to the secondary metabolites that are part of the chemical composition of propolis. The chemical composition of propolis can vary significantly from one source to another, depending on factors such as the geographical location where its components were collected, the type of vegetation growing in the area, and the season during which it was produced. Therefore, it is crucial to take into account the origin of propolis, as these variations in chemical composition may impact its efficacy and suitability for specific therapeutic applications [11, 12]. Understanding the content of propolis is essential in harnessing its potential as an antifungal agent, as different propolis sources may offer distinct advantages in addressing fungal infections [13, 14].

Currently, numerous scientific studies has been carried out on propolis, with different scientific approaches; in the particular case of propolis from Mexico, for example, a chemical comparison between propolis from different bee species from Yucatan was carried out, highlighting their terpenes composition [15]; the antiviral effect of propolis from Mexico against Coronavirus HCoV-229E has been tested in vitro, using human fibroblast lung cells, were experimental groups treated with propolis showed antiviral activity, attributed to the flavonoids as part of its chemical composition [16]; antiinflamatory and antioxidant effects of propolis from the North of Mexico has been tested in vivo, in a indomethacin-gastritis induced mice model, were propolis decreased mucosal damage, histological injuries and proinflamatory cytokine production on gastric ulcer injuries [17]; the ethanolic extract from propolis from Guanajuato, Mexico, showed cytotoxic effects against HeLa, SiHa and Caski ,cancer cell lines, besides antibacterial and antioxidant activities [18]; the ethanolic extract of propolis from Estado de México, México, decreased the cell infection and viral expression of the canine distemper virus [19]; and propolis samples from different regions of the country (Chiapas, Yucatán and Estado de México) inhibited lipid peroxidation damage and neutrophil migration [13].

The evaluation of the biomedical properties and chemical composition of propolis from different regions of Mexico is one of the main research lines of our research group; for example, previous assays and preliminary tests performed by our team have demonstrated that Mexican propolis from northern Mexico is rich in phenolic compounds, such as flavonoids, that are responsible for its antioxidant and anti-inflammatory properties that reduce the severity of diseases, as shown in a streptozotocin-induced diabetes experimental model and in indomethacin-induced gastritis [17, 20].

Notably, numerous studies have demonstrated that flavonoids have potential antimicrobial activities, including antifungal, antiviral and antibacterial activities [21].

Based on all of the above, we decided to evaluate the anti-*C. albicans* effect of Mexican propolis from Chihuahua, focusing on its antiproliferative effect, the inhibition of adhesion, and its morphological transition from yeast to hyphae, two of *C. albicans* most relevant virulence factors.

## Materials and methods

### Biological materials

Propolis samples (approximately 300 g) were collected in October 2018 from the apiary “Apiarios del Cielo” located in Ejido Concordia, Aquiles Serdán municipality, Chihuahua, Chihuahua, México. Ing. Martín Balcorta Baeza was the designed collector.

Propolis extract was obtained from 200.0 g of dry propolis through maceration (72 h) with 70% ethanol (1:3) using a rotary evaporator to distillate the solvent. The extract yield was 130.0 g (65.0%).

### Sensorial analysis of Mexican propolis from Chihuahua

A panel of eleven random evaluators participated in this study and estimated the organoleptic testing for the propolis sample.

The sample was given in disposable paper cups at 25 °C. The organoleptic testing consisted of a sensorial classification of color (yellow, dun, brown, reddish), smell (resinous, earthy), taste (waxy, spicy, balsamic) and consistency (soft, rigid).

### Sample preparation by silylation derivatization

A total of 5 mg of Chihuahua propolis ethanolic extract was prepared for gas chromatography by derivatization for 1 h at 100 °C with 50 µl pyridine + 75 µl bis-(trimethyl-silyl)trifluoroacetamide (BSTFA) including 1% trimethylchlorosilane (TMCS) in a sealed glass tube.

### Gas chromatography‒mass spectrometry of ethanolic extract samples

The ethanolic extract was analyzed by gas chromatography‒mass spectrometry using a Model 6850 chromatograph (Agilent Technologies, Santa Clara, CA, USA) coupled with a Model 5975C mass spectrometer (Agilent Technologies) and HP-5MS column (30 m × 0.25 mm, 0.25 μm Agilent Technologies).

The ethanolic extract sample (1 µl of the sample from a solution of 1 mg/ml) was injected in split mode. The initial temperature was 70 °C for 2 min followed by one heating ramp up from 15 °C min^−1^ to 290 °C and then maintained for 6 min. Helium was the carrier gas. The total analysis time was 31.0 min. The detected mass range was 35–600 m/z, the sample was ionized by electronic impact at 70 eV, and the ionization source temperature was 230 °C. The compounds were identified by comparison with the NIST version 8.0 library database (National Institute of Standards and Technology, Gaithersburg, MD, USA).

### Gas chromatography‒mass spectrometry of ethanol extract-derivatized samples

The ethanol extract-derivatized sample was analyzed by gas chromatography-mass spectrometry using a Model 6850 chromatograph (Agilent Technologies, Santa Clara, CA, USA) coupled with a Model 5975C mass spectrometer (Agilent Technologies) and HP-5MS column (30 m × 0.25 mm, 0.25 μm Agilent Technologies).

The derivatization mixture was evaporated, the derivatized sample was dissolved in HPLC hexane (High-Performance Liquid Chromatography solvent) (500 µl), and 1 µl of the sample was injected in split mode. An initial temperature of 100 °C was followed by a heating ramp up from 5 °C min^−1^ to 300 °C. Helium was the carrier gas. The total analysis time was 40.0 min. The detected mass range was 35–600 m/z, the sample was ionized by electronic impact at 70 eV, and the ionization source temperature was 230 °C. The compounds were identified by comparison with the NIST version 8.0 library database (National Institute of Standards and Technology, Gaithersburg, MD, USA).

### High-Performance Liquid Chromatography with diode array (HPLC-DAD) and High-Performance Liquid Chromatography -Mass Spectrometry (HPLC‒MS)

The ethanolic extract sample of Chihuahua propolis was analyzed in an HPLC-DAD system (Hewlett Packard, Agilent Technologies 1100 Wilmington, DE, USA) equipped with an 1100 diode array detector (DAD) operated with ChemStation A0903. The mobile phase consisted of methanol-acetonitrile-H_3_PO_4_-H_2_0 (25:25:0.1:50) under isocratic conditions for 35 min; this mobile phase was used because it gave the best resolution by injecting the standards of different flavonoids with which the database was built.

An Allsphere ODS-1 column (250 mm × 4.6 mm, with a particle size of 5 μm) at 269 bar pressure and a temperature range of 22–23 °C was used; the flow rate was 1 ml/min. A diode array detector (DAD) wavelength of 280 nm with a full scan of 200–400 nm was used. Compounds detected were identified according to comparison of the retention time and their absorption maxima (λ_max_) under ultraviolet light with those of the standards.

HPLC database standards included the following: kaempferol, catechin, pinocembrin, baicalein, naringenine, naringin, catechol, quercetin, luteolin, genistein, caffeine, apigenin, myricetin, chrysin, and acacetin. All standards were purchased from Sigma‒Aldrich (St. Louis, USA).

HPLC-ESI-TOF-MS was performed using an Agilent 1200 Infinity LC coupled to an Agilent 6230 TOF mass spectrometer with an Agilent Dual ESI Source (ESI SG1 4289023) and Mass Hunter Workstation Software, Version B.05.01, Build 5.01.5125.3, operating in negative ionization mode. The capillary voltage was 4000 V; the dry gas temperature was 250 °C; nitrogen was the dry gas at a flow rate of 6 l min^−1^; the nebulizer pressure was 60 psi; the fragmentor was 200 V; the MS range was 50-1300 m/z; and the MS acquisition rate was 1 spectrum/s.

The chromatographic separation was accomplished using an HPLC system (Infinity Series 1200, Agilent Technologies, Germany) equipped with a Kinetex 2.6 u, C18 100 Å column (150 × 2.1 mm) (Phenomenex, USA).

### Total phenolic content (TPC)

The concentration of total phenolic content (TPC) present in the ethanol extract of propolis was evaluated using Folin-Ciocalteu’s reagent, as described previously by Das et al. [22] and as reported previously by our team [23]. Briefly, a calibration curve of serial dilutions (0.00625 mg/ml-0.2 mg/ml) of gallic acid was created. Then, 0.5 mL of propolis extract (1 mg/mL) was mixed with 7.5 ml of distilled water and 0.5 mL of Folin-Ciocalteu’s reagent and allowed to stand at 22 °C for 5 min. Then, 1.5 mL of sodium carbonate (Na_2_CO_3_, 20%, w/v) was added, and the mixture was allowed to stand for another 90 min in the dark with intermittent shaking.

Propolis samples were tested in triplicate, and the absorbance was measured at 760 nm using a Multiskan SkyHigh Spectrophotometer (Thermo Fisher Scientific Inc. Singapore).

Finally, a linear regression analysis was performed, and sample absorbance was interpolated on the constructed calibration curve; the results were reported as milligrams of gallic acid equivalent per gram of extract (GAeq/g of extract).

### Total flavonoid content (TFC)

Quantification of total flavonoid content (TFC) was performed using the chloride colorimetric assay [24] as reported by our team previously [25]. Briefly, a calibration curve of serial dilutions (1-100 µg/ml) of quercetin was created. Propolis extract (0.2 mg/ml) dissolved in methanol was mixed with a solution of 2% aluminum trichloride (AlCl_3_) dissolved in HPLC-grade methanol. In an ELISA plate, 200 µL of the mixture was added, and the samples were incubated at room temperature for 10 min in the dark. The samples were tested in triplicate. The absorbance was measured at 415 nm with a Multiskan SkyHigh Spectrophotometer (Thermo Fisher Scientific Inc. Singapore).

A linear regression analysis was performed, sample absorbances were interpolated on the constructed calibration curve, and the results were reported as milligrams of quercetin equivalent per gram of extract (Qeq/g of extract).

### Antioxidant capacity

Propolis antioxidant capacity was determined by the 2,2-diphenyl-1-picrylhydrazyl (DPPH) reduction assay, as described previously by Okusa et al. [26]. The electron-donating capacity of the extract was calculated from the bleaching of the purple-colored DPPH solution dissolved in methanol. Ninety-six-well ELISA plates were filled with extract concentrations ranging from 1 to 100 µg/ml and 100 µM DPPH solution. Quercetin was used as a control, and the same concentrations of the extract were used. After 30 min of incubation in a dark room at 37 °C, the absorbance was measured at 517 nm with a Multiskan SkyHigh Spectrophotometer.

The antioxidant capacity values were determined according to the following equation:

$${\mathrm{AC}}_{50}=\;\lbrack(\mathrm{absorbance}\;\mathrm{of}\;\mathrm{control}\:-\:\mathrm{absorbance}\;\mathrm{of}\;\mathrm{sample})/\mathrm{absorbance}\;\mathrm{of}\;\mathrm{control}\rbrack\;\ast\;100$$

### Anti-*Candida albicans* activity of Mexican propolis from Chihuahua

#### Disk diffusion assays to test activity against *Candida albicans* strains

Chihuahua ethanolic propolis extract was tested on three strains of *Candida albicans*: a) a strain isolated from a clinical case donated by the Laboratory of Clinical Analysis of the FES-CUSI Iztacala (resistant to fluconazole: 25 µg Bio-Rad Marnes-la-Coquette France and ketoconazole 50 µg Bio-Rad Marnes-la-Coquette France); b) ATCC 14065 (resistant to fluconazole and ketoconazole); and c) CDBB-L-1003 (CINVESTAV, IPN, Mexico) (resistant to fluconazole and ketoconazole).

The anti-*Candida* activity was evaluated with the disk diffusion method, following the guidance of the CLSI. The yeasts were grown for 48 h at 37 °C in 10 ml of RPMI-1640 liquid medium. Cultures were adjusted at 1.0 × 10^6^ UFC/ml by counting the yeasts in a Neubauer chamber and making the necessary dilutions with RPMI-1640 liquid medium. The yeast suspensions were plated on Mueller-Hinton agar (2% glucose and 0.5 µg/ml methylene blue dye). Five mm diameter discs (Whatman no. 5) were impregnated with 10 µl of the propolis solution (final dose per disc: 4 mg). Discs containing ethanol served as the negative control, whereas discs with 25 µg of nystatin and 100 µg of amphotericin B were used as the positive control. The plates were incubated for 24 h at 36 °C, and the diameter of the growth inhibition zones (mm) was measured. The tests were performed in triplicate [27, 28].

### Broth microdilution assay

The 25% minimum inhibitory concentration (MIC_25_), 50% minimum inhibitory concentration (MIC_50_) and minimum fungicidal concentration (MFC) were determined by broth microdilution assay [29]. The yeasts were grown for 48 h at 37 °C in 10 ml of RPMI-1640 liquid medium. Cultures were adjusted at 1.0 × 10^3^ UFC/ml by counting the yeasts in a Neubauer chamber and making the necessary dilutions with RPMI-1640 liquid medium. The ethanolic propolis extract concentrations were in the range of 0.625-10 mg/ml. Microtubes were inoculated with 50 µl of 10^3^ UFC/ml yeast suspension in RPMI-1640 liquid medium. Inoculated plates were incubated at 36 °C for 48 h. After incubation, the size of the visible fungal growth was analyzed, and a sample was taken and grown in a Petri dish with Mueller-Hinton agar (2% glucose and 0.5 µg/ml methylene blue dye) to count colony forming units (CFU). The negative controls were microtubes with 36 µl of 70% ethanol. Each experiment was repeated at least three times [28, 29].

### Time-killing fungal kinetic assay

The effect on yeast growth was determined using the appropriate concentrations of the propolis ethanolic extract (MIC_25_, MIC_50_, and MFC). 100 µl of an initial inoculum ranging from 1-1.5 × 10^5^ UFC/ml were seeded in tubes with 10 ml of RPMI-1640 liquid medium. The samples were incubated at 36 °C. At 0, 3, 6, 24, 27, 30, and 48 h, volumes of 50 µl were seeded in the first division of a 3-division Petri dish with Mueller-Hinton agar (2% glucose and 0.5 µg/ml methylene blue dye); in the second division, a dilution of 1/100 was seeded, and finally, in the third division a dilution of 1/10,000 was seeded. An isotonic 0.85% sodium chloride solution was used to make the dilutions. Each experiment was repeated at least three times [30].

### Germ tube formation assay

To study the effect of the ethanolic extract of propolis from Chihuahua on the capability of *C. albicans* to change its morphology as one of its virulence factors, we promoted the yeast to hyphal transition by generating germ tube formation by stimulating *C. albicans* with fetal bovine serum (500 µl). A total of 50 µl of an inoculum of 1 × 10^6^ CFU/ml was added, and the cells were incubated at 36 °C for 3 h. After the incubation period, cells with evident germ tube formation were counted in an improved Neubauer chamber. Yeasts were counted as germinated if the germ tube was 3-fold larger than the original yeast. The assay was performed in triplicate, and the results are shown as the percentage of inhibition of germ tube formation [31, 32].

### Cell wall integrity assay

Calcofluor-white (Sigma‒Aldrich, St. Louis, MO, USA), a dye that binds to cellulose and chitin on the cell walls of fungi, and propidium iodide (Sigma‒Aldrich, St. Louis, MO, USA), a dye used to discriminate dead cells due to increased permeability on plasma membranes, were used to evaluate the activity of the ethanolic extract of propolis from Chihuahua.

The interaction of *C. albicans* ATCC 14065 with fetal bovine serum (500 µl) and propolis extract concentrations of 1 and 2 mg/ml was carried out for 3 h at 37 °C. Preparations for confocal microscopy consisted of equal volumes (10 µL) of *C. albicans* samples and both dyes.

Images were acquired using an SP8 LIGHTNING confocal microscope from Leica Microsystems (Wetzlar, Germany). Preparations were analyzed at a total magnification of 100×. Calcofluor-white dye was viewed at a wavelength of 450 nm, and propidium iodide was viewed at 580 nm. All experiments were performed in triplicate.

### RNA extraction and cDNA synthesis

The yeasts were grown for 48 h at 37 °C in 10 ml of RPMI-1640 liquid medium. Cultures were adjusted at 1.0 × 10^5^ UFC/ml by counting the yeasts in a Neubauer chamber and making the necessary dilutions with RPMI-1640 liquid medium. The concentrations of ethanolic extracts of propolis used were 1 mg/ml (CF_50_) and 2 mg/ml (CFM) and were incubated overnight at 36 °C. Total RNA was isolated from these cultures. An AllPrep kit (Qiagen, Hilden, Germany) was used. TURBO DNAse (Ambion, Carlsbad, CA, USA) was used to remove DNA (gDNA) from the purified RNA. RNA quality was checked by agarose gel electrophoresis at 80 V for 40 min, and the concentration was measured to estimate purity using a fluorometer (Thermo Fisher Scientific, Waltham, MA, USA). Single-stranded cDNA was synthesized using a SuperScript®III Reverse Transcriptase Kit (Thermo, Waltham, MA, USA) with oligo-dt.

### Quantitative real-time polymerase chain reaction (qRT‒PCR)

cDNA was used to amplify the *INT1* gene with the primers and conditions established [33]. β-actin was used as a housekeeping gene [34]. *INT1* gene expression was calculated according to E = Peff (-∆Ct), where Peff is the primer efficiency calculated using LinRegPCR [35]. Fold changes were calculated between the ratio expression of all conditions analyzed for three biological replicates.

### Statistical analysis

The mean and standard deviation of the experiments were determined. Analysis of variance (ANOVA) was performed to test for significant differences (*p* < 0.05) with Tukey’s honestly significant difference (HSD) multiple comparison test using the GraphPad Prism version 7 program.

## Results

### Sensorial analysis of Mexican propolis from Chihuahua

The specific color, smell, taste and consistency of propolis were unanimous between evaluators. The organoleptic properties of the propolis are shown in Table 1.

**Table 1:** Organoleptic characteristics of Mexican propolis from Chihuahua

Organoleptic characteristics	
Color	Dun
Smell	Resinous and earthy
Taste	Balsamic
Consistency	Rigid

### Chemical analysis of the propolis

The chemical composition of the Chihuahua propolis is shown in Tables 2, 3 and 4. In the analysis of the sample of ethanol extract and ethanol extract derivatized by GC‒MS (Tables 2 and 3), most of the identified compounds were different and, only pinostrobin chalcone and pinocembrin were detected in both samples. The analysis of the derivatized sample showed different carbohydrates.

**Table 2:** Compounds present in the Chihuahua propolis ethanolic extract identified by gas chromatography‒mass spectrometry

Compound	Retention time (min)	Abundance (%)
Benzoic acid	10.938	0.65
Pentadecanoic acid, 14-methyl-,methyl ester	23.052	4.65
Palmitic acid	23.497	3.14
2-Heptadecanone	25.300	3.77
Chrysin	26.118	2.38
Methyl n-hexadecyl ketone	27.603	2.49
Islandicin	27.715	4.17
1-(3-amino propyl)- azacyclotridecan-2-one	28.096	2.63
Pinostrobin chalcone	28.875	2.41
Pinocembrin	29.928	2.52

**Table 3:** Compounds present in the derivatized sample of Chihuahua propolis ethanolic extract identified by gas chromatography‒mass spectrometry

Compound	Retention time (min)	Abundance (%)
Benzyl methyl ketone	4.6425	0.28
D-Fructose	18.3328	0.49
Arabinofuranose	19.0385	2.28
Sorbopyranose	19.1283	5.19
D-Xylofuranose	19.4234	2.52
Glucopyranose	20.6359	6.53
6-O-methyl-beta-D-Glucopyranose	21.1491	4.60
Pinostrobin chalcone	29.2388	0.21
Pinocembrin	30.8234	0.69

**Table 4:** HPLC-DAD and HPLC‒MS analysis of the ethanolic extract of Chihuahua propolis

Name	Retention time (min		λ_max_ (nm)	Parent ion (m/z)[M-H]^−^	Relative error (ppm)
	HPLC-DAD	HPLC-MS			
Naringenin	4.913	23.152	230, 288	271.0642	10
Chrysin	14.060	nd	268, 312, 348	nd	nd
Pinocembrin	14.733	30.057	296, 334	285.0796	-12.7

*nd* Not detected

The HPLC-DAD analysis of the ethanolic extract sample showed 22 different compounds, and only three compounds matched our base data. The analysis of the ultraviolet spectrum of the other compounds detected showed that they correspond to the group of simple phenols and flavonoids. The HPLC‒MS analysis of the ethanolic extract sample showed 23 major compounds, of which only two compounds matched our base data (Table 4).

### Total phenolic content, total flavonoid content and in vitro antioxidant capacity of the ethanolic extract of Chihuahua propolis

Propolis ethanolic extract showed an adequate AC_50_ (Table 5), total phenolic content, and total flavonoid content according to the criteria of the Mexican norm for propolis quality standards (NOM-003-SAG/GAN-2017), which considers that propolis with an AC_50_ of less than 100 µg/ml, content of total phenols at least 5% and total flavonoids 0.5% minimum is acceptable.

**Table 5:** Total phenolic content (TPC), total flavonoid content (TFC) and antioxidant capacity of the ethanolic extract of Chihuahua propolis

	TPC (mg GAeq/g of extract)	TFC (mg Qeq/g of extract)	AC_50_ (µg/ml)
Chihuahua propolis	258	135.4	41.2
Quercetin			3.5

TPC is mg equivalents of gallic acid, and TFC is mg equivalents of quercetin

### Anti-*Candida albicans* activity of the ethanolic Mexican propolis extract from Chihuahua

Regarding the inhibition halos, there were no significant differences between the group treated with propolis and the group treated with amphotericin; the most effective inhibition halos were present in the group treated with nystatin (2-way ANOVA, Tukey’s multiple comparisons test p < 0.0001), while there was resistance against fluconazole and ketoconazole (Table 6).

**Table 6:** Anti-*Candida albicans* activity of the ethanolic extract of Chihuahua propolis

	Inhibition halos (millimeters)					
Strain	Propolis	Nystatin	Amphotericin	MIC_25_ (mg/ml)	MIC_50_ (mg/ml)	MFC (mg/ml)
*C. albicans* (cc)	10.5 ± 1.0	14.8 ± 0.5	11.5 ± 0.6	0.5	1.0	2.0
* C. albicans* (ATCC 14065)	11.8 ± 1.7	14.8 ± 0.5	11.3 ± 0.5	0.5	1.0	2.0
* C. albicans* (CDBB-L-1003)	10.0 ± 0.8	14.8 ± 1.0	11.8 ± 0.5	0.5	1.0	2.0

Filter paper disks whit 4 mg of propolis, nystatin 25 µg; amphotericin 100 µg. The three strains of *C. albicans* were resistant to fluconazole (25 µg) and ketoconazole (50 µg)

**Fig. 1:**
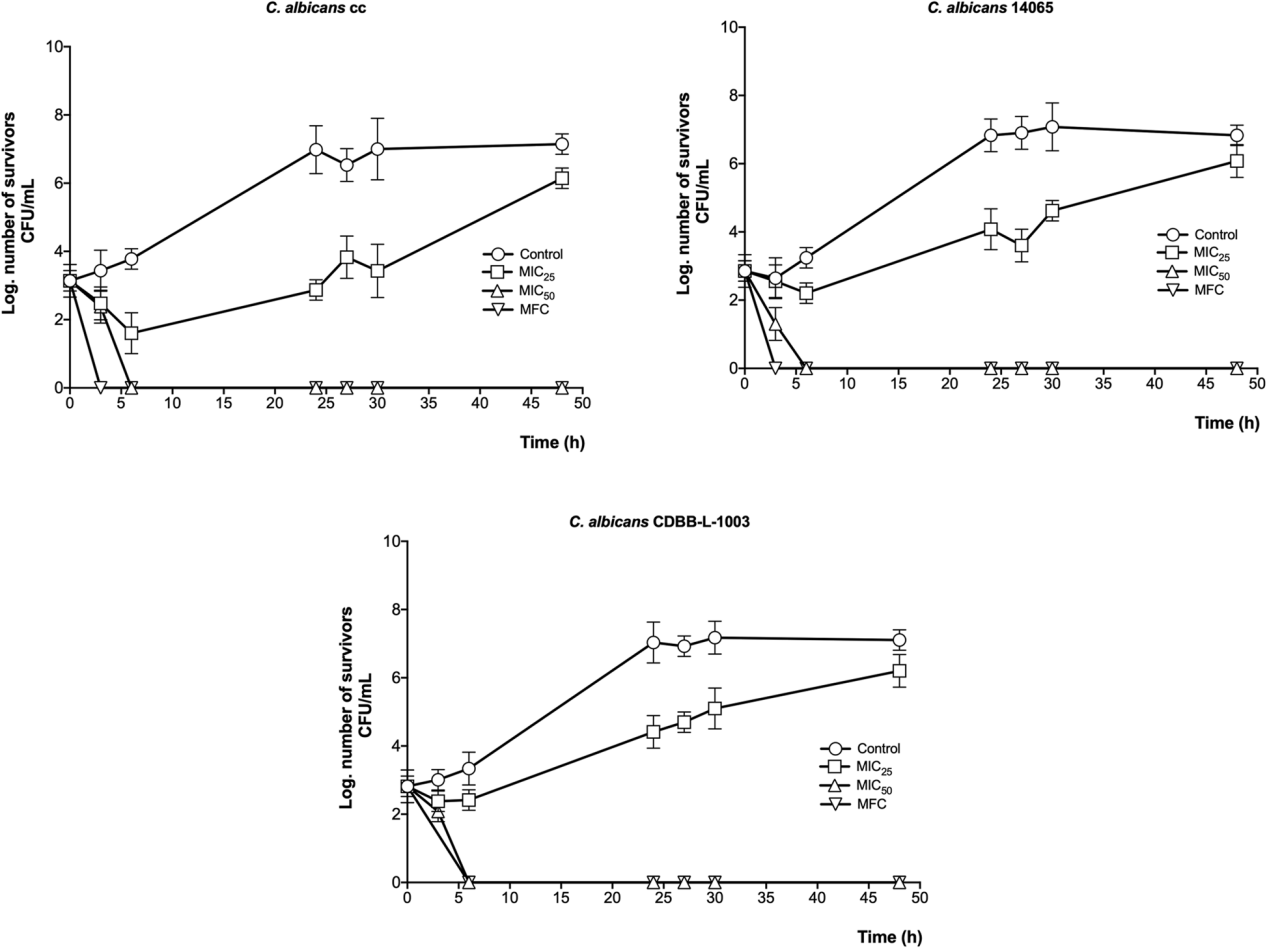
Time-killing fungal kinetic assay of the propolis ethanolic extract against the *C. albicans* clinical case, *C. albicans* ATCC 14065. *C. albicans* CDBB-L-1003. MFC = 2.0 mg/ml; MIC_50_ = 1.0 mg/ml; MIC_25_ = 0.5 mg/ml

Regarding the time-killing kinetic activity of the propolis ethanolic extract, a similar effect was observed in the three *Candida* strains; CF_25_ had fungistatic activity, while CF_50_ and CFM showed fungicidal activity (Fig. 1).

Regarding the inhibition of germ tube development, *C. albicans* ATCC 14065 was the strain most affected in germ tube formation; the CF_50_ (1.0 mg/ml) of the propolis ethanolic extract inhibited more than 60%, and CFM (2.0 mg/ml) completely inhibited germ tube formation (Table 7).

**Table 7:** Percentage of inhibition of germ tube formation by the propolis ethanolic extract

Inhibition of germ tube formation (%)							
Strain	0.06 mg/ml	0.125 mg/ml	0.25 mg/ml	0.5 mg/ml	1.0 mg/ml	2.0 mg/ml	CI_50_ mg/ml
*C. albicans* (cc)	28.15 ± 3.21	38.75 ± 5.24	51.15 ± 0.90	52.35 ± 1.19	54.98 ± 0.13	88.7 ± 4.39	0.30 ± 0.05
* C. albicans* (ATCC 14065)	36.93 ± 1.74	48.50 ± 1.12	51.48 ± 1.01	61.53.03 ± 2.25	63.23 ± 3.55	100.0 ± 0.00	0.18 ± 0.02
* C. albicans* (CDBB-L-1003)	32.73 ± 7.37	34.58 ± 7.37	45.2 ± 2.38	48.85 ± 1.67	51.98 ± 1.90	94.23 ± 3.85	0.47 ± 0.03

Propolis ethanolic extract concentration of 5 mg/ml inhibited 100% of germ tube formation and, killed all yeast used in the assay

**Fig. 2:**
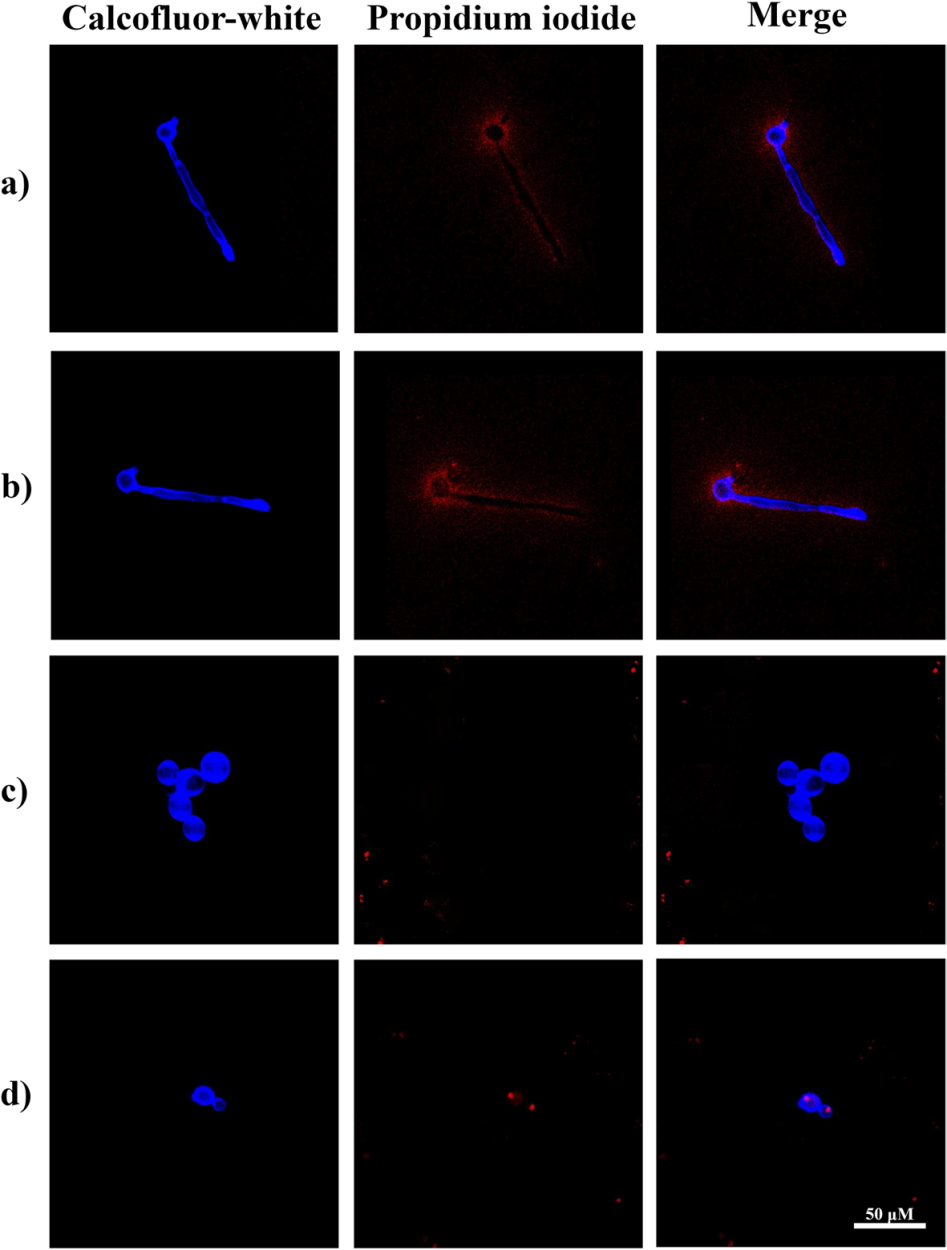
Activity of the ethanolic extract of propolis on the wall and membrane of *C. albicans* (ATCC 14065). The yeasts were stained with calcofluor-white and propidium iodide and visualized by confocal microscopy at 100× total magnification. Calcofluor-white dye was viewed at a wavelength of 450 nm, and propidium iodide was viewed at 580 nm. First observation column at 450 nm; second observation column at 580 nm; third observation column is the merge of the 2 wavelengths. **a** Control (fetal bovine serum 500 µl).; **b** negative control (with fetal bovine serum 500 µl and 28 µl 70% ethanol).; **c** ethanolic extract of propolis 1.0 mg/ml; **d** ethanolic extract of propolis 2.0 mg/ml

The staining with calcofluor-white and propidium iodide results showed that the ethanolic extract of propolis definitely affects the membrane of *C. albicans*. At a concentration of 1.0 mg/ml (Fig. 2c first column), the shape of the yeast was noticeably altered and germ tube formation did not occur; at a concentration of 2.0 mg/ml (Fig. 2d first column), how propidium iodide had noticeably penetrated and bound with the DNA and there was no development of the germ tube, indicating that the integrity of cell membrane was damaged (Fig. 2d second and third column). In the control (Fig. 2a) and negative control (Fig. 2b) groups at 450 nm, the germ tube was fully developed, and the blue fluorescence showed that the cell wall was not damaged; in these groups (second and third columns), the propidium iodide was left outside the cells, indicating that the membrane was intact.

**Fig. 3:**
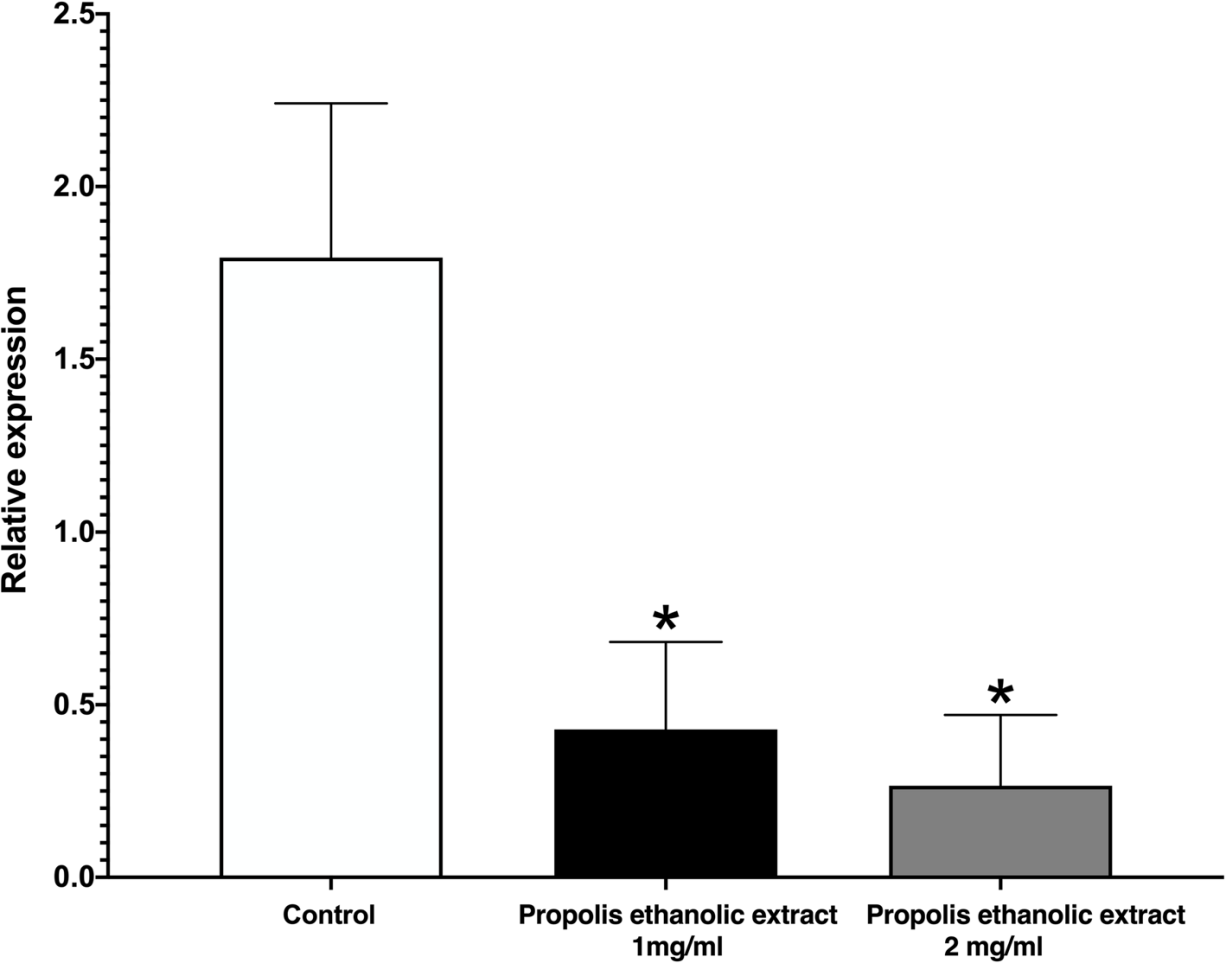
qRT‒PCR of the expression of the *INT1* gene of *Candida albicans* exposed to CF_50_ (1 mg/ml) and CFM (2 mg/ml). All values are expressed as the relative expression of the *INT1* gene ± SD. One-way ANOVA supported Tukey’s multiple comparisons test analysis and showed that both propolis ethanolic extracts at 1 mg/ml and 2 mg/ml had similar expression. * *p* < 0.05 compared to the control group

In the matter of *INT1* gene expression, the CF_50_ (the lower concentration used in this assay) was enough to inhibit more than 50% of this gene expression, meaning that the propolis affects *C. albicans* INT1p adhesin protein (Fig. 3).

## Discussion

Propolis is not the product of the hive to which the beekeeper focuses his attention; however, propolis has proven to be a product with important biomedical properties. These characteristics depend, to a great extent, on the location of the apiary and the plants that bees visit [36, 37].

The organoleptic characteristics of the ethanolic extract of propolis from Chihuahua (Table 1) are under the provisions of the Official Mexican Standard (2017) [38]. Regarding its chemical composition, GC‒MS analysis (Tables 2 and 3) revealed the presence of various compounds, such as fatty acids like pentadecanoic acid and palmitic acid, benzoic acid, some carbohydrates, and flavonoids, among others; these data coincide with other propolis analyses [39–41]. As expected, the HPLC analysis (Table 4) showed the presence mainly of phenols and flavonoids, compounds characteristic of propolis from different geographical areas [19, 39, 42].

Regarding the concentration of total phenols, total flavonoids, and average antioxidant capacity, the ethanolic extract of propolis from Chihuahua falls within the provisions of the Official Mexican Standard since it is established that Mexican propolis must contain at least 5% phenols (Chihuahua propolis 25.8%) and 0.5% flavonoids (Chihuahua propolis 13.54%), and the mean antioxidant capacity must be less than 100 µg/ml [38] (Table 5).

It is essential to mention that one of the problems with scientific research on propolis, is the variation in chemical composition from samples of propolis from different regions and countries around the world; in consequence, the biological properties of propolis may as well be variable [43] and may diminish the impact of scientific research. A helpful tool to solve this problem is the implementation of Official Standard Norms, like the Official Mexican Standard, (2017) that establish a series of characteristics necessary for a product to be considered as propolis, like its phenolic and flavonoid composition and thus, ensure a range of minimum biomedical properties.

Anti-fungal activity of propolis has been well documented in the literature [3], and it is known that the variation influences this biomedical property in the chemical composition of propolis [44]. Considering the chemical composition, antioxidant capacity, and a wide range of biomedical properties determined by our work team in the ethanolic extract of propolis from Chihuahua, including anti-bacterial and anti-inflammatory and its use in traditional medicine, we decided to evaluate its activity on *C. albicans* growth, adhesion and transition from yeast to hyphal form, two of its more important virulence factors. The ethanolic extract of propolis similarly inhibited the growth of all *C. albicans* strains tested (Table 6). However, regarding the activity on the growth curve, *C. albicans* 14065 was the most sensitive to the extract since MIC_25_ did not allow any rebound in its growth, as occurred with the clinical case strain at 27 h of sampling. The MIC_50_ and MFC showed fungicidal activity against all three *C. albicans* strains. These data are relevant since, for example, a sample of propolis from Brazil was shown to be only fungistatic, in addition to having greater values for MIC_50_ (1.675 mg/ml) and MFC (3.35 and 6.7 mg/ml) [45] than those reported here for propolis from Chihuahua. It is most likely that its antifungal activity against *C. albicans* is due a multitargeting involving several antifungal mechanisms [46], as the ethanolic extract of propolis from Chihuahua is a complex mixture of chemical compounds, highlighting its richness in flavonoids.

The transition of yeast to filamentous forms of *C. albicans* is an essential process for *C. albicans* virulence since it enables tissue invasion and biofilm formation, among others [47]. Notably, the ethanolic extract of Chihuahua propolis demonstrated an inhibitory effect on *C. albicans* changes from yeast to hyphae. After 3 h of interacting with yeasts at a concentration of 1 mg/ml, germ tube formation showed inhibition of more than 50%, and at 2 mg/ml, 100% of the development of the germ tubes was inhibited in the strain *C. albicans* ATCC 14065 (Table 7).

On the other hand, with calcofluor white and propidium iodide staining, it was shown that the ethanolic propolis extract also affects the integrity of the cell membrane. In addition to preventing the morphological change, there is also an increase in the permeability of the cell membrane, as the propidium iodide was already intercalated with the nucleic acids, which means that the dye was able to perfuse through the cell membrane (Fig. 2). This phenomenon is similar to other reports were using other cell viability dyes (Trypan blue, neutral red), it was observed that propolis from other regions affects the cell membrane of yeasts [45]. This damage observed in the integrity of the yeast cell membrane correlates with the fungicidal activity (MFC and CF_50_) of the propolis extract and the inhibition of the development of the germ tube.

Phenols and flavonoids present in the ethanolic extract of propolis are probably interacting with the structural elements of the cell wall, like proteins, chitin, and β-glucans, causing a loss of integrity, a decrease in rigidity and, consequently, a loss in the ability to resist osmotic pressure [46]; significantly, in addition to flavonoids, fatty acids detected as part of the chemical composition of the ethanolic extract of propolis from Chihuahua may be contributing to its antifungal activity, due to it has been shown that saturated fatty acids, like palmitic acid, can mimic the quorum sense molecule farnesol, interfering with the system and leading to inhibition of biofilm and hyphal formation [48], while unsaturated fatty acids, like pentadecanoic acid, can insert themselves into the lipidic bilayer of fungal membranes, compromising the membrane integrity and altering processes like the release of intracellular electrolytes and proteins, leading to cytoplasmic disintegration [49]. Figure 2c shows an example of these changes, where the yeasts lost their shape and their size increased. In the search for other possible mechanisms of action of propolis on the development of *C. albicans* and its virulence factors, and with a prophylactic approach for future research, it should be considered that the ethanolic extract of propolis from Chihuahua very likely can also be acting on the cell wall integrity of this yeast, like other works were propolis from Brazil was capable of altering the structure of the cell wall of *C. albicans* [45].

The *INT1* is a crucial gene that contributes to filamentous growth and encodes an adhesin (Int1p) that promotes the adherence of *C. albicans* to host cells or their specific ligands [47].

RNA/DNA and protein synthesis inhibition is well recognized as an antifungal target [50, 51]. Various reports have shown that some flavonoids, for instance, catechin, quercetin, kaempferol, naringenin, and gallic acid, can inhibit nucleic acid synthesis on different *C. albicans* strains [46]. The extract of this propolis significantly inhibited the expression of the *INT1* gene of *C. albicans* at the two concentrations tested, showing a higher inhibition value for CF_50_ (Fig. 3).; therefore, the extract of propolis inhibiting the RNA synthesis of INT1 gen and thus, the traduction of the adhesin Int1p. This effect can be well attributed to the synergic action of the flavonoids present in the propolis extract and their capacity to inhibit nucleic acid synthesis on *C. albicans*, diminishing RNA synthesis and expression of proteins [46], including important ones such as this adhesin.

In this research, it was found that the ethanolic extract of propolis from Chihuahua presented an adequate average antioxidant capacity, was fungistatic at a concentration of 0.5 mg/ml and fungicidal at a concentration of 1 mg/ml, inhibited the formation of the germ tube, damaged the integrity of the cell wall and membrane and inhibited the expression of the *INT1* gene, which codes for the synthesis of an essential protein in the yeast adhesion process to the host epithelium [52].

As mentioned earlier, propolis is a complex mixture of chemical compounds, including wax, resin, balsam, essential oils, pollen and plant primary and secondary metabolites, such as vitamins, terpenoids, phenolics, tannins and alkaloids [44]; one of the scientific problems that has received attention according to the study of propolis, is that, as a complex mixture, it is difficult to find a main component responsible of these biomedical effects, and it must be considered that propolis compounds are acting in synergism with multi-targeting strategy [43, 44, 53]; however, among all, it has been considered the presence of phenolic compounds, particularly flavonoids, as the responsible group for many of the broad spectrum of pharmacological activities, including the fungicidal activity against different *Candida* species, like *C. albicans*, *C. glabrata, C. pelliculosa, C. parapsilosis*, and *C. famata* [44]; flavonoids have shown to inhibit fugal growth with various underlying action mechanisms, such as plasma membrane disruption, by inhibition of ergosterol bioshyntesis, promotion of lipid peroxidation and inhibition of fatty acid synthase activity [46]; induction of mitochondrial dysfunction, by altering the mitochondrial electron transport chain and ATP production; inhibition of cell wall formation, by inhibition of β-glucans and chitin synthesis, which causes cell wall deformation and cell size reduction [46, 54]; cell division, by cell cycle arrest; protein expression, by inhibition of nucleic acid synthesis, causing deregulations in RNA/DNA synthesis; and the efflux mediated pumping system, which can lead to significant cell sensibility to drugs and activate apoptosis pathways [46, 54].

Flavonoids identified as part of the ethanolic extract, such as chrysin, have analgesic, anti-inflammatory, antibacterial, antiviral, and antioxidant effects [55–57]; pinocembrin has been shown to have antifungal activity by disruption of several critical cellular processes; such as damaging cell membranes and causing ionic leakage and ringworm [58, 59]; naringenin has antioxidant, anti-inflammatory and antiviral activity [59, 60]; and benzoic acid has been shown to have antifungal activity through interaction with nonspecific components in the cell membrane [61]. It is essential to mention that these are only a few flavonoids we managed to identify in this work, but it is very probable that the composition of flavonoids is more extensive and enhances propolis antifungal mechanisms; it could be boarded with a prophylactic approach. On the other hand, it is essential to note that in the chemical analysis of propolis samples from the same region [17, 20] and other regions [19], flavonoids have been identified that were also found in this propolis (naringenin, pinocembrin, chrysin). However, more studies are needed on Mexican propolis and its chemical characterization.

The complexity mentioned above of the chemical composition of propolis, although it may be considered problematic in some aspects of scientific research, like the identification of a single compound responsible for biomedical properties displayed, it also provides exciting approaches; for instance, it is well known that antifungal drugs, such as nystatin and amphotericin B, used in this research as positive controls, are only partially effective and may produce complications to host tissues [46, 62]. In contrast, natural products like propolis are low or no-toxicity therapeutic agents. Furthermore, it is essential to mention that both nystatin and amphotericin B bind to ergosterol and act at the cell membrane level, disrupting essential processes like endocytosis, cell division, membrane fluidity, and cell signalling [63]; however, RNA/DNA synthesis and protein traduction are unaltered; thus, propolis chemical complexity provides it with a multi-targeting strategy, enhancing the inhibition of *C. albicans* survival and replication by different pathways, as shown in this work.

Given the properties demonstrated for Chihuahua propolis, we propose that it is a candidate to be considered as an ideal antifungal agent, fulfilling the characteristics cited by Mazu et al., 2016 [61]; it presented broad spectrum inhibition of several strains of *C. albicans*, inhibited the growth of strains of filamentous fungi (*Fusarium moniliforme*, *Trichophyton mentagrophytes*, *Aspergillus niger*) (data not shown), displayed fungicidal activity, and had an effect on *C. albicans* ability to change from yeast to hypha and on its adhesion capacity; thus, the antifungal molecular targets of the propolis from Chihuahua must be very likely, at least in part, at the cell wall and cell membrane. In traditional medicine, propolis is administered orally, topically incorporated in a gel, or as a spray in the throat of the patient; also, it has been shown that it is not toxic. In addition to the above, the fact that this propolis inhibits the expression of the *INT1* gene and consequently the expression of the Int1p protein makes it an ideal antifungal since it has been shown that inhibition of INT1 gene in a murine model of systemic candidiasis, resulted in *C. albicans* loss of virulence [64].

## Conclusions

In this work, we demonstrated that Chihuahua propolis extract inhibits the proliferation of *Candida albicans* fungicidal activity; significantly, it also inhibits the development of the germ tube and the expression of the INT1 gene, two important virulence factors, probably by targeting some molecules present at the membrane and cell wall and destabilize cell dynamics.

Comparing the efficacy of propolis ethanolic extract with that of conventional antifungal drugs, such as nystatin, fluconazole, and ketoconazole, propolis extract was shown to show similar or better antifungal activity than conventional drugs in terms of inhibition halos. However, it is essential to note that more studies are needed to fully evaluate the potential of propolis as an alternative or complementary treatment for *Candida albicans* infections.

Using propolis in conjunction with conventional antifungal agents to treat fungal infections caused by *Candida* species offers several advantages in addressing resistant patterns among these fungi: Enhanced Antifungal Activity: propolis complements the action of conventional antifungal agents, providing a more robust response to *Candida* infections and helping overcome resistance. Multi-Target Approach: propolis targets *Candida* through multiple mechanisms, making it harder for the fungus to develop resistance. Reduced Risk of Cross-Resistance: Combining propolis with different antifungal agents reduces cross-resistance likelihood. Broad-Spectrum Coverage: propolis has broad-spectrum activity against various *Candida* species, ensuring coverage even for less responsive strains. Virulence Factor Inhibition: propolis inhibits *Candida* virulence factors, weakening the fungus ability to cause infection. Potential Synergistic Effects: propolis and antifungal drugs may synergize, leading to faster clearance of infections. Low Toxicity: propolis has minimal side effects, minimizing adverse reactions when used alongside antifungal agents. Alternative Treatment Option: propolis is an alternative when conventional antifungal agents are less effective due to resistance. However, it is crucial to base treatment decisions on scientific research, clinical trials, patient factors, and *Candida* species involved.

## Data Availability

All data generated or analyzed during this study are included in this published article.
